# Targeting Hsp27/eIF4E interaction with phenazine compound: a promising alternative for castration-resistant prostate cancer treatment

**DOI:** 10.18632/oncotarget.20469

**Published:** 2017-08-24

**Authors:** Hajer Ziouziou, Claudia Andrieu, Erik Laurini, Sara Karaki, Maurizio Fermeglia, Ridha Oueslati, David Taieb, Michel Camplo, Olivier Siri, Sabrina Pricl, Maria Katsogiannou, Palma Rocchi

**Affiliations:** ^1^ Inserm, UMR1068, CRCM, Marseille, France; ^2^ Institut Paoli-Calmettes, Marseille, France; ^3^ Aix-Marseille Université, Marseille, France; ^4^ CNRS, UMR7258, CRCM, Marseille, France; ^5^ Molecular Simulation Engineering (MOSE) Laboratory, DEA, University of Trieste, Trieste, Italy; ^6^ National Interuniversity Consortium for Material Science and Technology (INSTM), Research Unit MOSE-DEA, University of Trieste, Trieste, Italy; ^7^ Unit of Immunology Microbiology Environmental and Carcinogenesis (IMEC), Science Faculty of Bizerte, University of Carthage, Bizerte, Tunisia; ^8^ Aix-Marseille Université, CNRS, Centre Interdisciplinaire de Nanoscience de Marseille, UMR 7325, Marseille, France

**Keywords:** prostate cancer, Hsp27/eIF4E interaction, protein-protein interaction inhibition

## Abstract

The actual strategy to improve current therapies in advanced prostate cancer involves targeting genes activated by androgen withdrawal, either to delay or prevent the emergence of the castration-refractory phenotype. However, these genes are often implicated in several physiological processes, and long-term inhibition of survival proteins might be accompanied with cytotoxic effects. To avoid this problem, an alternative therapeutic strategy relies on the identification and use of compounds that disrupt specific protein-protein interactions involved in androgen withdrawal. Specifically, the interaction of the chaperone protein Hsp27 with the initiation factor eIF4E leads to the protection of protein synthesis initiation process and enhances cell survival during cell stress induced by castration or chemotherapy. Thus, in this work we aimed at i) identifying the interaction site of the Hsp27/eIF4E complex and ii) interfere with the relevant protein/protein association mechanism involved in castration-resistant progression of prostate cancer. By a combination of experimental and modeling techniques, we proved that eIF4E interacts with the C-terminal part of Hsp27, preferentially when Hsp27 is phosphorylated. We also observed that the loss of this interaction increased cell chemo-and hormone-sensitivity. In order to find a potential inhibitor of Hsp27/eIF4E interaction, BRET assays in combination with molecular simulations identified the phenazine derivative 14 as the compound able to efficiently interfere with this protein/protein interaction, thereby inhibiting cell viability and increasing cell death in chemo- and castration-resistant prostate cancer models *in vitro* and *in vivo*.

## INTRODUCTION

Prostate cancer (PC) represents the second cause of cancer mortality in men, mainly because patients relapse to castration-resistant prostate cancer (CRPC) two years after androgen ablation. The final treatment option is chemotherapy (docetaxel), but the median overall survival is only two/three months. Recently, cabazytaxel and Abiraterone has been approved as chemo- and endocrine-therapy agents but overall survival is only around 12 to 18 months [1, 2]. The actual therapeutic strategy to delay or prevent the emergence of the castration-refractory phenotype in advanced PC involves targeting genes activated by androgen withdrawal (for review [3]). Hsp27, a heat shock protein involved in pleiotropic cell functions [4] (for review [5]) is a highly overexpressed gene in CRPC [6, 7]. Aberrant expression of Hsp27 has been associated with tumor growth, resistance to hormonal therapy, cell death inhibition, and poor prognosis [6–9]. Silencing of Hsp27 expression by the use of antisense oligonucleotides (ASOs) or small interfering RNAs (siRNAs) increases apoptotic rates, induces tumor regression and enhances hormone- and chemotherapy in PC [6, 7]. Despite excellent results observed in relevant clinical trials ( http://www.oncogenex.ca/) the functional role of stress-induced Hsp27 in castration or chemotherapy-induced cell death remains poorly defined. The purpose of our work is to elucidate the pathways leading Hsp27 action in CRPC and to find new specific therapeutic targets and treatment strategy for CRPC that would have limited toxicity for normal tissues. Using two-hybrid experiment, we previously found that Hsp27 plays a major role in the protein translational initiation process [10]. These data led us to investigate the protein synthesis initiation pathway, a prerequisite for cell growth and proliferation. Thus, by northern and western blot analysis we found that Hsp27 down-regulation decreased eIF4E expression only at the protein level, without affecting mRNA content. The cytoprotection afforded by Hsp27 overexpression was attenuated by eIF4E knockdown using specific eIF4E siRNA. Co-immunoprecipitation and co-immunofluorescence experiments confirmed that Hsp27 co-localizes and interacts directly with eIF4E. Hsp27-eIF4E interaction decreases eIF4E ubiquitination and proteasomal degradation. By chaperoning eIF4E, Hsp27 protects the protein synthesis initiation process to enhance cell survival during cell stress induced by castration or chemotherapy. Forced over-expression of eIF4E induced resistance to androgen-withdrawal and docetaxel treatment in the prostate LNCaP cells line *in vitro*. In aggregate, these findings identified Hsp27 as a modulator of eIF4E and established a potential mechanism for the eIF4E-regulated cell death after androgen ablation and chemotherapy. Accordingly, targeting Hsp27-eIF4E interaction may serve as a therapeutic option in advanced PC [10].

In humans, a variety of tumor types have been previously described to exhibit elevated levels of eIF4E, including advanced PCs [11]. eIF4E overexpression and translation initiation is involved in malignant transformation and chemo-resistance *in vitro* and *in vivo* and thus represents an important target for cancer therapy [11]. Many approaches over the years have been used to try to inhibit eIF4E functions, particularly the use of small molecule inhibitors that can disrupt the eIF4E/eIF4G interaction, the use of cap analogs to directly target the eIF4E cap-binding site, or ASOs that have been proven to be efficient in reducing the expression level of eIF4E and are tested in many clinical trials in prostate cancer patients [12].

Our aim was to disrupt this mechanism involved in castration-resistant progression of prostate cancer. Towards this goal, we worked on the identification of the interaction site of Hsp27/eIF4E. In order to find a potential inhibitor of this interaction, we performed a screening of compounds by BRET experiments and found one candidate, a derivative of phenazine, compound 14, that was not a DNA intercaling agent and inhibited Hsp27/eIF4E interaction leading to cell viability inhibition and increase of apoptosis of castration-resistant prostate cancer cells.

## RESULTS

### eIF4E interacts with the C-terminal region of Hsp27 leading to chemo- and hormone-resistance and absence of Hsp27 phosphorylation partially inhibits this interaction

**Figure 1:**
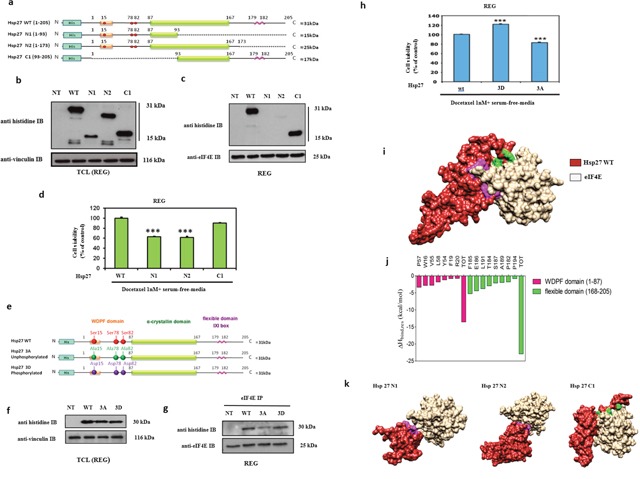
Modeling of Hsp27 with eIF4E and phosphorylation status of Hsp27 reveal that Hsp27 C-terminal domain and phosphorylation are essential for protein binding and loss of this interaction increases cell chemo and hormone-sensitivity **(a)** Schematic representation of Hsp27 wild type (WT) and truncated mutant forms of Hsp27 (N1, N2, and C1). Mutants N1 and N2 correspond to two different variants of the N-terminal region of Hsp27 protein (residues 1-93, and 1-173, respectively), whereas mutant C1 corresponds to the Hsp27 C-terminal region, containing the last 113 protein residues (residues 93-205). The full-length WT Hsp27 sequence was used as control. No endogenous Hsp27 expression REG cells transiently transfected or not (NT) with plasmids containing WT Hsp27 and N1, N2 and C1 and proteins were extracted for: **(b)** Western Blot analysis of histidine and vinculin protein levels from total cell lysates (TCL) and **(c)** Western Blot analysis of histidine and eIF4E protein levels after immunoprecipitation (IP) using anti-eIF4E antibody **(d)** MTT quantification of REG cells viability. **(e)** Schematic representation of Hsp27 wild type (WT) and phosphorylation mutants of Hsp27 (3A and 3D) used in this study. In the former case, the 3A mutant of Hsp27 was specifically constructed by replacing the serine residues 15, 78 and 82 with alanine; concomitantly, the 3D Hsp27 isoforms was obtained by replacing the same serine residues with aspartic acid. **(f)** Western Blot analysis of histidine and vinculin protein levels from total cell lysates (TCL) and **(g)** Western Blot analysis of histidine and eIF4E protein levels after immunoprecipitation (IP) using anti-eIF4E antibody. **(h)** MTT quantification of REG cells viability was performed on cells transiently transfected with plasmids containing WT Hsp27 and phosphorylation mutants of Hsp27 prior to treatment with docetaxel in serum-free media (a condition mimicking androgen deprivation) *** P≤0.001. **(i)** Overall view of an equilibrated MD snapshot of the Hsp27 WT/eIF4E complex. The proteins are visualized by their van der Waals surfaces, colored as follow: Hsp27 WT, firebrick; eIF4E, khaki. The amino acids of Hsp27 mainly involved in binding eIF4E are highlighted as follows: purple, residues belonging to the WDPF domain; green, residues belonging to the flexible domain. See text for details. **(j)** Per residue enthalpic contribution to WT Hsp27 binding with eIF4E. Only those Hsp27 amino acids affording a meaningful contribution to protein-protein formation contributing (ΔH_bind,res_ < -0.80 kcal/mol) are shown for clarity. From this analysis, it further appears that the Hsp27 α-crystallin domain is practically ineffective in Hsp27/eIF4E binding since residues belonging to this Hsp27 region display a negligible contribution to protein-protein binding enthalpy. **(k)** Overall view of equilibrated MD snapshots of the eIF4E in complex with Hsp27 N1, N2 and C1 truncated mutant isoforms. In each panel, the proteins are visualized by their van der Waals surfaces, colored as follows: Hsp27s, firebrick; eIF4E, khaki. The amino acids of Hsp27 mainly involved in binding eIF4E are highlighted as follows: purple, residues belonging to the WDPF domain; green, residues belonging to the flexible domain. Interestingly, the overall structure of Hsp27 N1 is *de facto* strongly affected by the loss of the α-crystallin domain, which plays a major role in the overall correct chaperon folding required for effective binding to eIF4E.

Hsp27 deletion mutants were used to determine the region of the heat shock protein that interacts with eIF4E (Figure 1a). The results showed that eIF4E interacts only with the Hsp27 C1 mutant, while protein/protein interaction was inhibited when Hsp27 was truncated in its C-terminal part (N1 and N2 mutants) (Figures 1b-1c). To evaluate how the Hsp27/eIF4E interaction is involved in chemo-resistance, cells were transfected with the Hsp27 deletion mutants prior to docetaxel treatment in combination with androgen deprivation in rat colon carcinoma cancer cell line (REG) cells that have no endogeneous expression of Hsp27. The analysis of cells viability showed that cells sensitivity to chemotherapy (docetaxel) and androgen withdrawal (serum free media) increased in the presence of the N1 and N2 mutants (Figure 1d). Thus, our data confirmed that loss of Hsp27/eIF4E interaction *via* truncation of Hsp27 C-terminal region restores chemo- and hormone- sensitivity in cancer cells.

To evaluate the effects of Hsp27 phosphorylation on its interaction with eIF4E Hsp27 mutants corresponding to constitutively unphosphorylated (3A) or phosphorylated (3D) forms were used (Figure 1e). The results showed that the interaction of eIF4E with the phosphorylated form of Hsp27 (3D mutant) was similar to the one observed with the WT protein, while partial inhibition of the interaction was found with the unphosphorylated form (3A mutant) (Figure 1f–1g) showing that phosphorylation regulates the interaction. To understand the implication of Hsp27 phosphorylation on cell drug resistance, transfected cells were treated with docetaxel in serum free media. The analysis of cell viability showed that the unphosphorylated 3A mutant of Hsp27 restored sensitivity to cell death driven by docetaxel and androgen withdrawal, while no effect on cell viability was observed in the case of the constitutively phosphorylated mutant mimics (3D) (Figure 1h). The same results have been obtained in LNCaP prostate cancer models (data not shown). These results demonstrate that Hsp27 phosphorylation partly regulates Hsp27/eIF4E interaction and confirm that loss of this interaction can induce treatment-sensitivity.

To rationalize these results at a molecular level, first extensive molecular dynamics (MD) run was carried out on the WT Hsp27/eIF4E complex (Figure 1i). The retrieved inter-protein binding free energy ΔG_bind_ value of -11.86 kcal/mol (Table 1) revealed a good affinity between the two polypeptides, in agreement with the experimental evidences. A per-residue binding enthalpy (ΔH_bind,res_) analysis [13, 14] allowed to determine which Hsp27 residues are mainly involved in this interaction (Figure 1j). Accordingly, we found that WT Hsp27 binds the initiation factor mainly by exploiting the initial residues of the domain WDPF (W=tryptophan, D=aspartic acid, P=prolin and F=phenylalanine) and the final residues of the flexible domain in the C-terminal region, these last providing a favorable contribution to the binding two times larger than that afforded by the amino acids of the N-terminal part (Figure 1k).

**Table 1:** Binding free energies (ΔG_bind_) and binding free energy differences (ΔΔG_bind_) for the Hsp27 WT, N1, N2 and C2 truncated mutants in complex with eIF4E. (ΔΔG_bind_=ΔG_bind(WT)_- ΔG_bind(truncated)_)

Complex	ΔG_bind_(kcal/mol)	ΔΔG_bind_(kcal/mol)
Hsp27 WT	-11.86 ± 0.24	-
Hsp27 N1	-3.06 ± 0.27	-8.80
Hsp27 N2	-6.05 ± 0.23	-5.81
Hsp27 C1	-9.99 ± 0.25	-1.87

The same computational approach was applied to the binding of the three truncated forms of Hsp27 (i.e., N1, N2, and C1) to the eIF4E protein. As expected, the ΔG_bind_ values of the truncated forms were all less favorable compared to the WT protein (Table 1). Yet, a specific trend could be clearly discerned. Indeed, the presence of the C-terminal part still endow the Hsp27 C1 mutant with good eIF4E binding capability, inducing only a small decrease in binding affinity with respect to the WT (ΔG_bind(C1)_ = -9.99 kcal/mol). On the other hand, the Hsp27 N2 isoform is still able to maintain a minimal affinity against the eIF4E through its WDPF residues; however, the absence of the C-terminal region leads to a strong reduction of the interaction surface (Figure 1h) and this, in turn, reflects into a loss of almost 6 kcal/mol in ΔG_bind_ (Table 1). Lastly, the presence of the sole WDPF domain in the N1 Hsp27 mutant form allows the formation of a network of very weak interactions with eIF4E, reflecting in a plummet of ~9 kcal/mol in the relevant value of ΔG_bind_ (Table 1, Figure 1i). In aggregate, our *in silico* results confirmed that the most important interactions involved in binding of the Hsp27 protein with the initiation factor eIF4E are provided by its C-terminal part, strictly in agreement with the co-immunoprecipitation experiments.

### Screening of chemical compounds using BRET assay identify that phenazine#14 disrupt Hsp27/eIF4 interaction

**Figure 2:**
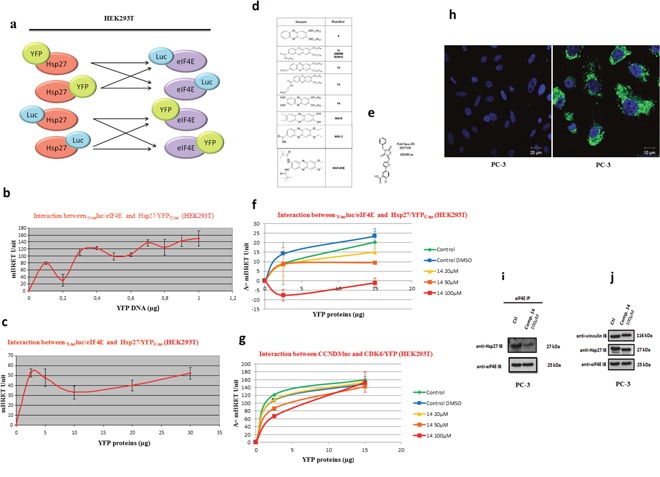
Validation of Hsp27/eIF4E interaction by BRET in whole living cells and in cells extracts and revelation of the chemical compound 14 as a specific inhibitor Hsp27/eIF4E by BRET screening **(a)** To by-pass technical parameters that could prevent us from measuring a BRET signal (presence and/or position of the reporter proteins that could disrupt the Hsp27/eIF4E interaction, physical distance between Rluc and YFP), the interaction between these two proteins with all possible couple combinations (n=8) of plasmids were tested. These couples of plasmids were transfected separately in HEK293T cells at different concentrations in order to find the ideal ratios and obtain the BRET signal. For the construction of BRET plasmids, we merged each gene (Hsp27 and eIF4E) with Rluc or YFP in N-terminal (N-ter) or C-terminal (C-ter) part. After, we tested the interaction between these two proteins with all the possible couple combinations of plasmids, on BRET on living cells or cells extracts. **(b)** Hsp27/eIF4E interaction in BRET experiment was investigated in HEK293T cell extracts. The principle was similar to BRET assay in living cells; except that the plasmids were transfected separately in HEK293T cells and that the couple combinations were tested *in vitro* by mixing proteins extracted from these transfected cells. HEK293T cells were co-transfected with 0.2 μg of BRET donor plasmid _N-ter_luc/eIF4E, and 0 to 1 μg of BRET acceptor plasmid Hsp27/YFP_C-ter_. The empty vector (pEYFP-C) was used to equalize DNA amounts to 1.2 μg in each sample. The reading of optic density was performed after the addition of coelenterazine in order to obtain the BRET signal. **(c)** HEK293T cells were transfected separately with a BRET donor plasmid _N-ter_luc/eIF4E or BRET acceptor plasmid Hsp27/YFP_C-ter_. Δ=(YFP/Luc-YFP_0_/Luc_0_)*1000. **(d)** Derivatives of phenazines that have been described to have an anti-tumor activity as well as a structure similar to inhibitors of eIF4E/eIF4G interaction. **(e)** Compound 4E2RCat, which is described in literature to be an inhibitor of the eIF4E/eIF4G interaction. **(f)** HEK293T cells were transfected separately with a BRET donor plasmid _N-ter_luc/eIF4E or BRET acceptor plasmid Hsp27/YFP_C-ter_. Total proteins were extracted from cells and used for BRET assay: 1 μg of lysate containing a BRET donor and 0 to 15 μg of lysate containing a BRET acceptor were pre-incubated separately with different concentrations (0, 20, 50 and 100μM; green, yellow, orange and red lines respectively) of compound 14 during 2h. As control experiment, cell extracts were pre-incubated with DMSO alone, at the higher concentration (1%) we used to dilute compound (control DMSO, blue line). Donor and acceptor were mixed for 30 min and the reading of optic density was performed after the addition of coelenterazine in order to obtain the BRET signal. **(g)** The same experiment was performed with another couple of protein: CCND3/luc and CDK6/YFP (4μg of lysate containing BRET donor was used). Δ=(YFP/Luc-YFP_0_/Luc_0_)*1000. **(h)** PC-3 cells were treated at 100μM with compound 14 (right panel, Bar=10 μm) and DMSO (left panel, Bar = 20μm) as control. Auto-fluorescence of compound 14 (green) and staining of the nucleus by DAPI (blue) was observed. PC-3 cells were treated with DMSO (control) or compound 14 during 48h and proteins were extracted for: **(i)** Western Blot analysis of Hsp27, eIF4E protein levels after immunoprecipitation (IP) using eIF4E rabbit antibody or IgG rabbit (control) **(j)** Western Blot analysis of Hsp27, eIF4E, Vinculin and protein levels from total protein extracts.

To further visualize Hsp27/eIF4E interaction and in search for eventual inhibitors of this interaction, BRET assay in living cells and in cell extracts were performed using different combination of constructs (Figure 2a) of Hsp27 and eIF4E with YFP (Yellow Fluorescent Protein) or luc (luciferase) bound either to the N- or to the C-terminal region of both proteins (see SI). We confirmed the interaction between fusion protein Hsp27/YFP_C-ter_ and fusion protein _N-ter_luc/eIF4E in HEK293T living cells (Figure 2b). BRET signal with the same couple of proteins was obtained as previously described for BRET experiment on living cells (Figure 2c). Next, to identify potential inhibitors of the Hsp27/eIF4E interaction, a screening of chemical compounds on cell extracts was carried out. We performed our screening using phenazine derivatives that were previously shown to have an anti-tumor activity in pancreatic and prostatic cell lines [15, 16] and structure similar to inhibitors of the eIF4E/eIF4G interaction (Figure 2d). At the same time, we tested the compound “4E2RCat” reported in literature as an inhibitor of the eIF4E/eIF4G interaction, in order to see if it also inhibited the Hsp27/eIF4E interaction [17, 18] (Figure 2e). Of all compounds tested, only 14 revealed a dose-dependent inhibitory effect on the Hsp27/eIF4E interaction (Figure 2f). Other phenazine derivatives, as well as 4E2RCat, showed no significant inhibition (not shown). To see if this inhibition was specifically due to Hsp27/eIF4E interaction rather than to non-specific protein degradations, 14 was tested on an irrelevant couple of proteins, CCND3/Luc and CDK6/YFP. The result showed no inhibition of this interaction by 14, supporting the specificity of Hsp27/eIF4E interaction inhibition (Figure 2g). This screening allowed us to highlight 14 as the first compound that potentially inhibits Hsp27/eIF4E interaction.

To confirm the fact that the phenazine-derivative 14 can inhibit the Hsp27/eIF4E endogeneous interaction in cells, we performed immunofluorescence and co-immunoprecipitation experiments. Our results performed in androgen-independent prostate cancer PC-3 cells demonstrated that 14 is located in the cell cytoplasm (Figure 2h) prevents the Hsp27-eIF4E interaction (Figure 2i) without any effect on proteins expression levels (Figure 2j).

### Computational studies confirm the inhibition of Hsp27/eIF4E interaction by the phenazine 14 and reveal the potential the molecular mechanism of the inhibition

**Figure 3:**
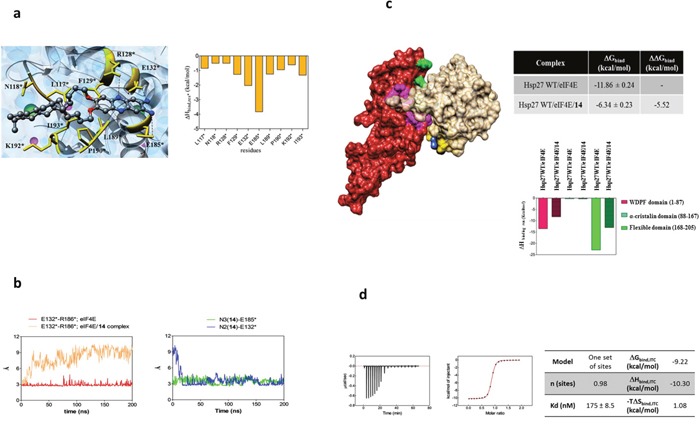
Molecular modeling and isothermal titration calorimetry (ITC) reveal the mechanism of Hsp27/eIF4E interaction inhibition by compound 14 **(a)** (Left panel) Zoomed view of an MD equilibrated snapshot of the 14 in complex with eIF4E. Specifically, the ligand is docked between a α-helix of eIF4E protein spanning residue L117*-E132* and a structurally hybrid region from E185* to I193*. The ligand is portrayed in ball-and-stick representation and colored by element (C, gray; N, blue; O, red). The main protein residues involved in compound binding are depicted as gold sticks and labelled. Transparent light blue spheres represent water oxygen atoms, while chlorine and sodium ions and counterions are shown as green and purple spheres, respectively. Hydrogen atoms are omitted for clarity. (Right Panel) Per residue binding enthalpy decomposition for eIF4E residues mainly involved in binding with 14. Only those Hsp27 amino acids affording a meaningful contribution to protein-drug formation (ΔH_bind,res*_ < -0.50 kcal/mol) are shown for clarity. The network of stabilizing hydrophobic interaction involves the two C_12_ alkyl chains of the 14 and the side chain of the protein residue L117*, N118*, F129*, L189*, P190*, K192*, and I193*. The two amine substituents of 14 are engaged in two persistent polar interactions with the carboxylic side chain of E132* and E185*. In addition, a further stabilizing interaction via a weak hydrogen bond between the side chain of R128* and a nitrogen atom of the phenazine ring is detected. **(b)** (Left panel) MD simulation distance between the charged side chains of E132* and R186* in the eIF4E alone (red line) and in the eIF4E/14 complex (salmon line). In the eIF4E free protein, the two α-helixes spanning residues Q122*-I138* and R173*-G188*, respectively, are persistently stabilized by the presence of permanent interaction points between the side chain of their residues. In particular, a strong polar interaction between the charged side chain of the amino acids E132* and R186* is detected. As shown, the Average Dynamics Length (ADL) of this interaction between the two involved atoms is 2.96 Å, and its persistence is verified along the entire MD run. Conversely, the same distance progressively increases during the first part of the simulations and finally settles around 9Å when the eIF4F is simulated in presence of the ligand. The reason for this behavior can be explained by that fact that E132* is engaged in a polar interaction with 14, as demonstrated by the complementary trend exhibited of the corresponding distance between the carboxylic moiety of E132* and the amine group of 14 shown in the right panel (MD simulation distance between the N2 nitrogen atom of 14 and the charged side chain of E132* (blue line), and between the N3 nitrogen atom of 14 and the charged side chain of E185* (green line). **(c)** (Left panel) Overall view of an equilibrated MD snapshot of the Hsp27 WT/eIF4E/14 complex. All molecules are visualized by their van der Waals surfaces, colored as follow: WT Hsp27, firebrick; eIF4E, khaki; 14, navy blue. The amino acids of Hsp27 mainly involved in binding eIF4E are highlighted as follows: purple, residues belonging to the WDPF domain; green, residues belonging to the flexible domain. The residues of eIF4E mainly involved in binding with 14 are depicted in gold. (Upper right panel) Binding free energies (ΔG_bind_) and binding free energy differences (ΔG_bind_) for the WT Hsp27/eIF4E (plain filled columns) and the WT Hsp27/eIF4E/14 (patterned filled columns) complexes. (Lower right panel) Comparison of the clustered per residue enthalpic contribution to binding for WT Hsp27/eIF4E and WT Hsp27/eIF4E/14 complexes. **(d)** ITC experiment of 14/eIF4E binding: (left) raw data; (middle) titration curve; (right) binding thermodynamics parameters.

The BRET assay established that compound 14 inhibits the interaction between the WT Hsp27 protein and the initiation factor eIF4E. We reasoned that this disruption could be caused by the binding between the initiation factor and 14, with the subsequent alteration of the protein structure. The final effect could ultimately translate in the modification of the interaction surface between eIF4E and WT Hsp27, which, in turn, implies impairing the corresponding energy of binding. To verify this hypothesis, a plausible binding cavity for 14 in a region of the protein that belongs to the interacting surface of the Hsp27/eIF4E complex was identified (Figure 3a). Then, (MD) simulation of the resulting eIF4E/14 complex was carried out, and the MM/PBSA results yielded a good binding affinity of the molecule for the protein (ΔG_bind_ = -9.05 kcal/mol), in agreement with its inhibitor activity demonstrated in the BRET assay.

The specific eIF4E/14 binding mode was again analyzed by a per-residue decomposition of the enthalpic component of the free energy of binding ΔH_bind,res*_. The collected data (Figure 3a) revealed that the eIF4E/14 complex is stabilized by three main interaction types: i) a network of stabilizing hydrophobic interaction involving the two C_12_ alkyl chains of the 14; ii) two persistent polar interaction anchoring both amine substituents of 14 to the protein, and iii) a weak hydrogen bond engaging a nitrogen atom of the phenazine ring (Figure 3a).

The stable complex between 14 and eIF4E had also an important consequence from the protein structure viewpoint, in that it reflects in an overall arrangement of the eIF4E structure, specifically in the region involved in the protein-protein interface with Hsp27 (Figure 3b). To corroborate this assumption, the same computational approach was applied to the WT Hsp27/14/eIF4E complex, and the inhibitory effect of 14 was quantified. Actually, the presence of the ligand leads to a drastic reduction of the binding interactions between WT Hsp27 and the initiator factor, with a loss of about 5.5 kcal/mol in the corresponding calculated free energy of binding (Figure 3c).

Titration of 14 into the protein solution by Isothermal Titration Calorimetry (ITC) confirmed the binding results obtained by our computational procedure (Figure 3d). Indeed, the affinity of 14 for eIF4E is in the nanomolar range (K_D_=175 nM). Moreover, the corresponding ΔG values of -9.22 kcal/mol (obtained from the fundamental relationship ΔG = RT lnK_D_), is in outstanding agreement calculated by MM/PBSA computational approach (-9.05 kcal/mol, see above). For the thermodynamic viewpoint, the process is largely enthalpy driven (ΔH = -10.3 kcal/mol), while entropy variation slightly disfavors binding (-TΔS=1.06 kcal/mol). Finally, the molar ratio identified from the number of ligands per protein (*n*) is very close to 1 (*n* = 0.974), definitely supporting a 1:1 stoichiometry for the 14/eIF4E complex.

### Hsp27/eIF4E inhibition by phenazine 14 restores treatment sensitivity of androgen-independent prostate cancer models *in vitro* and *in vivo*

**Figure 4:**
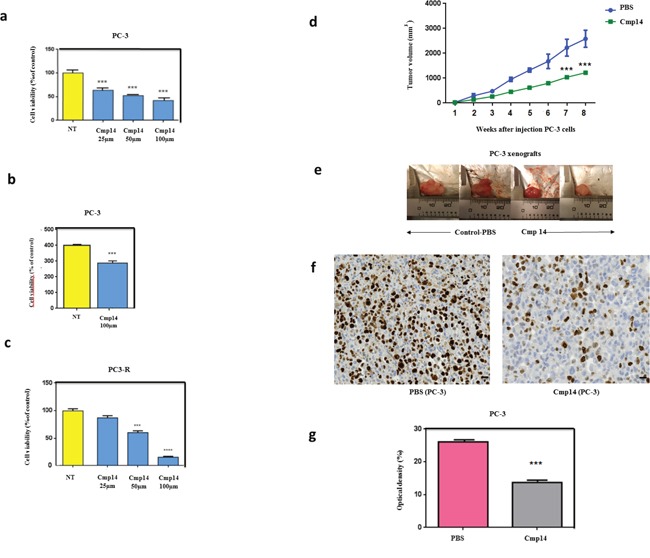
Compound 14 inhibits cell viability and increases apoptosis *in vitro* and *in vivo* Cells viability using MTT assay **(a)** was performed on PC-3 non treated (NT) or treated cells with compound 14 (a) at different concentrations (25, 50, 100μM) during 48h. Cell death quantification (SubG0 phase) using flow cytometry **(b)** was performed on PC-3 cells non treated (NT) or treated with compound 14 at 100μM during 48h. Cell viability using MTT assay was also performed on PC-3-docetaxel resistant cells non treated (NT) or treated with compound 14 **(c)** at different concentrations (25, 50, 100μM) during 48h. **(d)** PC-3 cells were subcutaneously implanted in Node Scids by injection of 10 ×10 ^6^ cells in the right flank of animals. When tumors reached 100^mm3^, mice were randomized in two groups that received twice a week an intra-peritoneal injection of PBS (control n = 6, blue) and phenazine 14 (n = 8, green) (1 mg/kg) for 8 weeks. Tumor volume was measured once weekly and calculated by the formula length x width x depth x 0.5236. Compound 14 reduced significantly PC-3 tumor volume by up to 50%. During the entire treatment period, all mice treated with PBS and 14 did not show any abnormal behavior, and no significant alteration of mice body weight was observed. **(e)** Photographs of PC-3 harvested tumors from mice that received i.p. compound 14 or control-PBS after an 8-week treatment **(f)** Ki-67 IHC staining of tumor tissues to assess tumor cells proliferation. **(g)** Distribution of tissue Ki-67 immunostaining intensity (measured as average optical density) according to the tumor treated with PBS and Compoud#14. Error bars represent the SE, **, P ≤0.01 and ***, P ≤ 0.001 by Statview software.

In order to evaluate the effect of the Hsp27/eIF4E interaction inhibition in prostate cancer model, we treated androgen-independent (PC-3) and docetaxel-resistant (PC3-DR) cells with different concentrations of phenazine 14. The inhibition of Hsp27-eIF4E interaction by 14 decreased cells viability in a dose-dependent manner (Figure 4a) and increased cell death in androgen-independent PC-3 model (Figure 4b). More interestingly, derivative 14 restored chemo-sensitivity in docetaxel-resistant induced PC-3^DR^ cells [19] (Figure 4c). Encouraged by the nontoxic, promising *in vitro* results obtained with compound 14, *in vivo* experiments were conducted to assess its effect on tumor growth. Figure 4d shows that 14 (green line) significantly reduced androgen-independent PC-3 tumor growth (***P ≤ 0.01) by up to 50% compared to PBS treatment (blue line). AT killing, tumor volume from animals treated with compound 14 were smaller than those harvested from PBS treated group (Figure 4e).

Immunohistochemical study analysis (Figure 4f) demonstrated that the proliferation index level (Ki-67) of compound 14 treated groups was significantly lower than that of control group (PBS), indicating lower cell proliferation in tumors and hence higher antitumor activity of this molecule. Optical density (OD) that correlates with the antigen expression was next measured. We found that the mean of Ki67 expression was significantly increased in tumors treated with PBS compared with tumors treated with 14 (26.09 ± 0.17 and 13.7 ± 0.18, p < 0.0001) (Figure 4g). These results confirmed the efficacy of the phenazine-derivative 14 in decreasing tumor proliferationinandrogen-independent PC-3 models.

## DISCUSSION

Development of CRPC is an inevitable progression of PCs after androgen ablation. At this stage, treatment options are very limited. Until recently, the chemotherapeutic agent docetaxel represented the treatment of choice after castration resistance emerged, prolonging the mean life span of patients for 2.9 months [20]. Overexpression of anti-apoptotic proteins, mRNA splicing events, gene fusions, loss of expression of tumor suppressor genes, post-transcriptional modification using miRNA, and epigenetic alterations are all hallmarks of CRPC [3]. Hsp27 has numerous cytoprotective functions and is involved in cell migration, cytoskeleton architecture, cell growth, differentiation, and tumor progression [21]. Despite the promising clinical trial results of Hsp27 inhibitor (OGX427, Apatorsen), long-term inhibition of the survival protein Hsp27 might be accompanied by cytotoxic effects due to the role of this protein in several physiological processes [22]. To avoid this problem, an alternative therapeutic strategy consists of using compounds that disrupt specific protein-protein interactions (PPIs) between Hsp27 and partners that mediate its cytoprotective effects [10, 21, 23].

In our previous investigations, we showed that Hsp27 interacts directly with eIF4E. Hsp27/eIF4E interaction decreases eIF4E ubiquitination and proteasomal degradation. By chaperoning eIF4E, Hsp27 appears to protect the protein synthesis initiation process to enhance cell survival during cell stress induced by castration or chemotherapy [10]. In this study, we sought to further characterize important facets of the interaction between Hsp27 and eIF4E. First, using both co-immunoprecipitation (Figure 1) and BRET assays (Figure 2), we demonstrated that the interaction between Hsp27 and eIF4E may involve the C-terminal domain of Hsp27, and that loss of this interaction restores chemo- and hormone-sensitivity of PC. Next, we determined that phosphorylation of Hsp27 enhances its interaction with eIF4E (Figure 1f–1g). Finally, we identified and characterized the first compound, 14 able to inhibit the Hsp27/eIF4E interaction (Figure 3).

To evaluate the effects of Hsp27 phosphorylation on its interaction with eIF4E, we used phosphorylated (3D) and non-phosphorylated (3A) forms to Hsp27. By immunoprecipitation we showed that eIF4E interacts with the phosphorylated forms of Hsp27, while only a partial interaction inhibition with the non-phosphorylated form of Hsp27 (Figure 1g) was detected. Structure and function studies indicate that the chaperone properties of Hsp27 correlate with the ability of the protein to form large oligomers, which are mediated by the non-phosphorylated form of Hsp27 [24, 25]. On the other hand, the triple mutant phosphorylated (3D) mimic is capable of forming at most tetramers and has less chaperone activity than the wild type protein [26].

Zoubeidi et al. have shown that phosphorylated Hsp27 displaces Hsp90 from androgen receptor thus taking its place, which allows transport of the receptor in the nucleus with subsequent activation and transcription of target genes. This interaction further avoids MDM2/AR binding and promotes MDM2 ubiquitination and degradation by the proteasome [27]. Our work indicates that Hsp27 has a similar role on eIF4E, protecting it from degradation by the ubiquitin/proteasome system and allowing maintenance of protein synthesis. The hypothesis of the “sorting” function of this protein is reinforced by the fact that Hsp27 seems to be selective with respect to targets to be degraded. For example, it acts on p27 ^kip1^ but has no action on other cell cycle proteins such as cyclin A, E, D1 or β-catenin [28, 29].

Another observation drawn from this study is that when only the N-terminal domain of Hsp27 is expressed it does not seem to interact strongly with eIF4E. The C-ter part of Hsp27 (C1 mutant) and the loss of this interaction (N1 and N2 mutants) are both involved, at least in part, in the increase of treatment-sensitivity of the cells (Figure 1b–1c). Thus, different portions of the Hsp27 molecule may be responsible for mediating the response to different stimuli. For example, the C-terminal domain may be largely responsible for the response to stress, whereas the N-terminal could be responsible for modulating hormonal signaling [30]. Another study indicates that the C-terminal of Hsp27 retains its flexibility during the interaction with dithiothereitol and suggests a highly flexible C-terminal in mammalian sHSPs is required for full chaperone activity [31].

It has been shown that the C-terminal phosphorylation of Hsp70 and Hsp90 acts as a switch for regulating co-chaperone binding and indicates that cancer cells possess an elevated protein folding environment by the concerted action of co-chaperone expression and chaperone modifications [32]. Literature data indicate that the chaperone-like activity of small heat shock proteins is dependent on the properties of the C-terminal extension and deletion [31, 33].

The overexpression of Hsp27 in cancer tissues is correlated with anti-cancer drug resistance, which makes Hsp27 a promising molecular target for drug development [8, 10]. Our present results demonstrate that compound 14 has a dose-dependent inhibitory effect on the Hsp27/eIF4E interaction (Figure 2f) in a BRET assay. Accordingly, 14 could represent an efficient and selective new anticancer compound, so far being the only phenazine derivative able of inhibiting the Hsp27/eIF4E interaction.

BRET assay constitutes an efficient methodology to study PPIs under native conditions compared with other screening assays performed using soluble fragments of the target protein. Our actual BRET assay demonstrated the capacity of compound 14 to disrupt Hsp27/eIF4E interaction. By analogy, the BRET approach was previously used with success in order to design and synthetize inhibitors able to disrupt Mcl/Bim interaction in ovarian carcinomas [34]. These inhibitors have the ability to dissociate Mcl-1 from Bim in whole living cells and acquired sensitivity ovarian cancer cells to Bcl-X and increased cell death.

Recently, other phenazine derivatives (2, 3-dialkoxyphenazines, e.g., compound **11**) were classified as anticancer agents. Indeed, these compounds were tested *in vitro* on human pancreatic cells lines (MiaPaCa-2) proving that these compounds appeare to be more cytotoxic than gemcitabine. *In vivo* results demonstrated an activity comparable to standard drug but at 10 times lower concentration [15].

As shown in Figure 4d, administration of free compound 14 moderately retarded tumor growth at 1mg/kg dosage, which was further supported by the immunohistochemical (IHC) analysis and quantification to evaluate tumor cell proliferation. Figure 4f reveals that the Ki-67 level of compound 14 treatment group was much lower than those of the alternative treatment group (PBS), indicating lower cell proliferation in tumors and hence higher antitumor activity of 14. In this study, we managed to determine the site interaction between two proteins considered as therapeutic targets in PC. Indeed, eIF4E is considered to be a proto-oncogene and its overexpression causes tumorigenic transformation of fibroblasts [35], while the overexpression of Hsp27 causes the resistance of tumor cells to treatment. The rationale of targeting Hsp27/eIF4E interaction is that long-term inhibition of two proteins separately may be toxic for the cells. Indeed, Hsp27 is a multifunctional protein involved in several biological processes and long inhibition of eIF4E leads to deregulation and malfunction of the translation initiation mechanism disrupting the process of cell transformation.

This work also allowed to better characterize the Hsp27/eIF4E interaction and demonstrated specific inhibition of this interaction by the phenazine-derivative 14. Indeed, the treatment of cells with 14 inhibited proliferation of androgen-independent cells *in vitro* and *in vivo* and restored docetaxel sensitivity in docetaxel-resistant PC cell. In conclusion, the phenazine-derivative 14 could represent an interesting therapeutic option to restore hormone- and chemo-sensitivity in late stage of the disease where there is no therapeutic alternative. We are currently working to improve solubility and biodisponibilty of this phenazine 14 compound by using nucleolipids nanovectors in order to be tested in further clinical trials.

## MATERIALS AND METHODS

### Cell lines and cell culture conditions

The human androgen-independent cell line PC-3 (American Type Culture Collection, USA) were maintained in DMEM (PC-3) media (Invitrogen, France) supplemented with 10% fetal bovine serum (FBS). The rat colon carcinoma cancer cell line REG was provided by Dr Carmen Garrido (INSERM U866, Dijon, France) and maintained in F10 medium (Invitrogen) supplemented with 10% FBS. The Human Embryonic Kidney cell line HEK293T (American Type Culture Collection, USA) was maintained in Dulbecco's Modified Eagle's Medium (Invitrogen), supplemented with 10% FBS. All cell lines were cultivated at 37°C in 5% CO_2_. PC-3DR-docetaxel resistant cell line [19] was kindly provided by Dr Martin Gleave (Vancouver Prostate Cancer Center) and was maintained in culture as previously described.

### Transient transfections of cells

Cells were transfected with the FuGENE HD Transfection Reagent (Promega, France) the day after seeding with 10μg DNA Hsp27 deletion mutant or Hsp27 phosphorylation mutant plasmids (see Hsp27 mutant plasmids) or BRET plasmids (see BRET section), as previously described [36].

### Immunoprecipitation

Cleared lysates with adjusted protein concentration (Pierce BCA Protein assay, Thermo Fisher scientific, France) were used for immunoprecipitation with 8 μl (1/50) of rabbit anti-eIF4E antibody (Cell Signaling, Ozyme, France) ON at 4°C as previously published [36]. Precisely, immune complexes were precipitated after 1 hour incubation with 30 μl of TrueBlot anti-rabbit Immunoglobulin beads (eBiosciences, Paris, France). The complexes were resuspended in protein sample buffer (Bio-Rad) and boiled for 5 minutes before western blot as described before. We used the rabbit TrueBlot anti-rabbit IgG secondary antibody (eBiosciences) to reveal the western blot.

### Western blot

Western blot (WB) was performed with 1/3000 mouse anti-polyHistidine antibody (Sigma-Aldrich, USA), 1/1000 rabbit anti-eIF4E antibody (Cell Signaling, Ozyme), 1/2500 anti-mouse IgG HRP conjugate antibody (Promega, France), 1/1000 anti-rabbit Trueblot IgG HRP conjugate antibody and 1/1000 anti-mouse Trueblot IgG HRP conjugate antibody (eBiosciences), 1/5000 anti-rabbit IgG HRP conjugate antibody (Santa Cruz Biotechnology, Germany), 1/5000 rabbit Hsp27 antibody (Assay Designs, FranceLoading levels were normalized using 1/2000 mouse anti-vinculin antibody (Sigma-Aldrich). Re-blot Plus Mild Solution (Millipore, France) was used for membrane stripping during 9 min at RT.

### *In vitro* cell viability assay

Cells were transiently transfected the day after seeding with Hsp27 deletion or phosphorylation mutant plasmids as described above, or treated with 14. Transfected cells were then treated with docetaxel (Sanofi-Aventis, France) in serum-free media (mimicking androgen withdrawal *in vitro*) for 24h. Cell viability was assessed using 3-(4, 5-dimethylthiazol-2-yl)-2, 5-diphenyl tetrazolium bromide (MTT) assay for REG, PC-3 and PC-3^RD^ cells, as previously described [36]. Each assay was performed in triplicate.

### Statistical analysis for viability assays

Statistical analysis was performed using the GraphPad Prism program (GraphPad Software, San Diego, USA). All data are mean ± SEM. Significance of differences was assessed by a two-tailed Student's t-test. *P ≤ 0.05 was considered significant, with **P ≤ 0.01 and ***P ≤ 0.001.

### Confocal microscopy

PC-3 cells were seeded into a 12-well plate containing cover glasses treated with FBS, at a density of 100 000 cells/well. 24h later, cells were treated with compounds (14, DMSO) at 100μM as indicated above. After 48h of treatment, cells were washed with PBS1X and fixed with formaldehyde 4% (Thermo Fisher Scientific, France) during 15’ at RT. Further extensive washing was performed with PBS1X before mounting the cover glasses on glass slides by using Prolong Gold anti-fade reagent with DAPI (Life Technologies, France). Glass slides were allowed to dry in the dark at RT for 24h, and cover glasses were then immobilized with nail polish. Fluorescent images of compound 14 (absorption; 452nm, emission; 478nm) and DAPI (absorption; 350nm, emission; 450-490nm) were captured with a Zeiss 510 META fluorescence confocal microscope plan 40X/1.4 (Le Pecq, France).

### Flow cytometry

Flow cytometry of propidium iodide-stained nuclei was performed as described previously [36]. Briefly, PC-3 cells were seeded into 10-cm dishes at a density of 125 0000 cells/well. 24h later, cells were treated with compounds 14 at 100μM as indicated above. DNA content was determined by flow cytometry using a LSRII SORP (Becton Dickinson, France) machine. Rates of cells death were then measured using FlowJo software (Tree Star, Inc.).

### Assessment of *in vivo* tumor growth

10^6^ PC3 cells were inoculated in the flank region of 2-week-old athymic male mice (NSG). Tumors were measured weekly and their volume was calculated by the formula length×width×depth×0.5236. When tumors reached 100 mm^3^, mice were randomly selected for treatment with PBS (control) or 14 alone. Each experimental group consisted of 6 control mice and 8 mice treated with 14. Phenazine 14 was tested at its highest solubility (1mg/ml). Injection lasted 8 weeks with two injections per week. Data points were expressed as average tumor volume levels ±SE.

### Immunohistochemistry

3 μm paraffin sections of tumors were dried overnight at room temperature. Before antibody staining, the slides were first incubated for 1.5 h at 65 °C, then incubated for 20 min at 95 °C with EnVision FLEX Target Retrieval Solution (low pH, pH 6) (K8005; Dako UK Ltd.), followed by pretreating with Epitope Retrieval Solution (containing detergent; K5207; Dako UK Ltd) for 30 min at room temperature to unmask binding epitopes. After blocking of endogenous peroxidase activity with Dako EnVision FLEX Peroxidase- Blocking Reagent SM801 (ready to use) (K8000, K8002, K8023; Dako UK Ltd.) for 5 min, the slides were washed thoroughly in wash buffer FLEX (Dako UK Ltd). After one wash in FLEX buffer, the slides were incubated with a FLEX Monoclonal Mouse Anti-Human Ki-67 (clone MIB-1, IR62; Dako UK Ltd.) for 1 h at room temperature. After two more washes in FLEX buffer, Dako EnVision FLEX/HRP SM802 (K8000; Dako UK Ltd.) was added for 20 min at room temperature. After two final washes with FLEX buffer, the staining was visualized by adding diaminobenzidine (Dako UK Ltd.) for 10 min at room temperature. The slides were washed well in FLEX buffer and counterstained with EnVision FLEX HEMATOXILIN SM806 (K8008; Dako UK Ltd.) for 5 min, then washed once with FLEX buffer, then with water, and then dehydrated, cleared, and mounted with aqueous mounting media (LEICA AUTOSTAINER JUNG XL). Positive and negative controls were performed with each batch of slides.

### Quantification of immunolabeling

A comparative quantification of immunolabeling in all tissues types was performed using Ki67 antibody. In each tumor section, the staining intensity (optical density) per unit surface area was measured with an automatic image analyzer (Motic Images Advanced version 3.2, Motic China Group Co., China) in 5 light microscopic fields per section, using the ×40 objective. Delimitation of surface area was carried out manually using the mouse of the image analyzer. For each positive immunostained section, one negative control section (the following in a series of consecutive sections) was also used, and the optic density of this control section was taken away from that of the stained section. From the average values obtained (by the automatic image analyzer) for each tumor, the means ± SEM for each tumor (PBS and 14) were calculated. The number of sections examined was determined by successive approaches to obtain the minimum number required to reach the lowest SEM. The statistical significance between means of the tumors group's samples was assessed by the Fisher exact test and the one-way ANOVA test at p≤0.05 (GraphPad PRISMA 5.0).

## SUPPLEMENTARY MATERIALS FIGURE


