# Correction: Targeting Hsp27/eIF4E interaction with phenazine compound: a promising alternative for castration-resistant prostate cancer treatment

**DOI:** 10.18632/oncotarget.25683

**Published:** 2018-06-19

**Authors:** Hajer Ziouziou, Claudia Andrieu, Erik Laurini, Sara Karaki, Maurizio Fermeglia, Ridha Oueslati, David Taieb, Michel Camplo, Olivier Siri, Sabrina Pricl, Maria Katsogiannou, Palma Rocchi

**Affiliations:** ^1^ Inserm, UMR1068, CRCM, Marseille, France; ^2^ Institut Paoli-Calmettes, Marseille, France; ^3^ Aix-Marseille Université, Marseille, France; ^4^ CNRS, UMR7258, CRCM, Marseille, France; ^5^ Molecular Simulation Engineering (MOSE) Laboratory, DEA, University of Trieste, Trieste, Italy; ^6^ National Interuniversity Consortium for Material Science and Technology (INSTM), Research Unit MOSE-DEA, University of Trieste, Trieste, Italy; ^7^ Unit of Immunology Microbiology Environmental and Carcinogenesis (IMEC), Science Faculty of Bizerte, University of Carthage, Bizerte, Tunisia; ^8^ Aix-Marseille Université, CNRS, Centre Interdisciplinaire de Nanoscience de Marseille, UMR 7325, Marseille, France

**This article has been corrected:** The proper listing of author names is given below:

**Hajer Ziouziou^1,2,3,4,7^, Claudia Andrieu^1,2,3,4^, Erik Laurini^5,6^, Sara Karaki^1,2,3,4^, Maurizio Fermeglia^5,6^, Ridha Oueslati^7^, David Taieb^1,2,3,4^, Michel Camplo^6^, Olivier Siri^8^, Sabrina Pricl^5,6^, Maria Katsogiannou^1,2,3,4^ and Palma Rocchi^1,2,3,4^**

Original article: Oncotarget. 2017; 8:77317-77329. https://doi.org/10.18632/oncotarget.20469

