# Supplementary material for: Targeting Hsp27/eIF4E interaction with phenazine compound: a promising alternative for castration-resistant prostate cancer treatment
Source: Oncotarget. 2017 Aug 24;8(44):77317–29. doi: 10.18632/oncotarget.20469 (PMC5652782; doi:10.18632/oncotarget.20469)
Supplement: Supplementary file 1 [file oncotarget-08-77317-s001.pdf]

Targeting Hsp27/eIF4E interaction with phenazine compound: a promising alternative for castration-resistant prostate cancer treatment

SUPPLEMENTARY MATERIALS

Bioluminescence resonance energy transfer (BRET) assay

Construction of BRET plasmids

Hsp27 or eIF4E genes alone or with kozak sequence upstream were first inserted into the pCR8/GW/TOPO plasmid (Invitrogen). Kozak sequence was added each time the gene was found in N-ter in the final plasmid. For the insertion into pCR8/GW/TOPO plasmid, genes were first amplified by PCR using Phusion enzyme (Finnzymes, Thermo Fisher Scientific, Illkirch, France) and the following BRET primers: Hsp27: Forward=5'-ATG ACC GAG CGC CGC GTC CC-3', $Forward_{kozak}$= 5'-GCC ACC ATG ACC GAG CGC CGC GTC CC-3', Reverse=5'-CTT GGC GGC AGT CTC ATC GG-3', eIF4E: Forward=5'-ATG GCG ACT GTC GAA CCG GAA AC-3', $Forward_{kozak}$=5'- GCC ACC ATG GCG ACT GTC GAA CCG GAA AC-3', Reverse=5'-AAC AAC AAA CCT ATT TTT AGT GGT GG-3'. Then, dATP with Go Taq enzyme (Promega, Charbonnieres, France) were added at the ends of the gene (PCR product) to allow matching between gene and plasmid. Genes were then purified from the PCR product with the Wizard SV Gel and PCR Clean-Up System kit (Promega). The insertions into plasmids were realized by TOPO reaction according to manufacturer's instructions (Invitrogen). Competent bacteria "MACH 1" (Invitrogen) were transformed using TOPO reaction products and the clones we obtained were analysed by PCR with Go Taq enzyme (Promega). Positives clones were amplified by mini-culture and DNA was extracted with Wizard Plus SV Minipreps DNA Purification System kit (Promega), and sent for sequencing (M13 Forward primer) (GATC, Mulhouse, France). The second step was gene transfer from pCR8/GW/TOPO intermediate plasmids to destination plasmids containing luciferase and YFP by LR Gateway recombination reaction using LR clonase according to manufacturer's instructions (Invitrogen). The destination plasmids pRLuc-C, pRLuc-N, pEYFP-N and pEYFP-C were received from Nelson Dusetti (UMR 1068, Centre Recherche Cancérologie Marseille, France). Competent bacteria "MACH 1" (Invitrogen) were transformed using LR reaction products. The clones obtained were amplified by mini-culture and DNA was extracted with the Wizard Plus SV Minipreps DNA Purification System kit (Promega). Enzymatic digestion by BsrGI (Promega) was performed in order to identify positives clones that were then amplified by maxi-culture and DNA was extracted using the PureYield Plasmid Midiprep System kit (Promega), and sent for sequencing (BRET primers) (GATC). For each gene (Hsp27, eIF4E) four plasmids with luciferase or YFP in N-terminal or C-terminal part were constructed. Constructs were checked by western blot, fluorescence visualization, and luminescence reading.

BRET in living cells

HEK293T cells cultured in 12-well culture plates (400 000 cells/well) were co-transfected with 0.2 µg of BRET donor plasmid $_{N\text{-}ter}$luc/eIF4E, and 0 to 1 µg of BRET acceptor plasmid Hsp27/$YFP_{C\text{-}ter}$. The empty vector (pEYFP-C) was used to equalize DNA amounts to 1.2 µg in each sample. After 24h, cells were harvested and distributed in 96-well white microplates (≈40 000 cells/well). On the following day, the cell-permeable Rluc substrate coelenterazine-h (Interchim, Montluçon, France) diluted in phosphate buffered saline (PBS) 1X was added in culture medium to a final concentration of 5 µM, and cells were incubated 15 min at 37°C before reading. Readings were done using a LB 941 Tristar reader (Berthold France SA, Thoiry, France), with signal detection in the 470-490 nm (donor) and 520-540 nm (acceptor). BRET signal represents the BRET ratio of the Rluc and EYFP expression constructs compared to the BRET ratio for the Rluc expression construct alone. To assess signal variation, the BRET values were determined by using the following equation, expressed in milli-BRET unit (mBretU):

$$\left[\left(\frac{530\,nm\,acceptor\,signal}{480\,nm\,donor\,signal}-E_0\right)\times 1000\right]$$

where E_0 corresponds to the ratio 530 nm acceptor signal/480 nm donor signal obtained with the Rluc construct alone in the same experiment. All experiments were performed at least three times with comparable results.

BRET in cell extracts

HEK293T cells were transfected separately with a BRET donor plasmid $_{N\text{-}ter}$luc/eIF4E or BRET acceptor

plasmid Hsp27/YFP$_{C-ter}$. After 48h, total cellular proteins were extracted following manufacturer's instructions of CelLytic NuCLEAR Extraction Kit (Sigma-Aldrich) and stored at -80°C. 1 µg of lysate containing a BRET donor $_{N-ter}$luc/eIF4E (or 4µg for CCND3/luc) was mixed with 0 to 30 µg of lysate containing a BRET acceptor Hsp27/YFP$_{C-ter}$ (or CDK6/YFP) and with lysis buffer in a 96-well white plate. After 30 min of incubation at RT, coelenterazine-h (Interchim) was added to a final concentration of 5 µM in lysis buffer, just before reading. For chemical compounds screening, cellular extracts containing BRET donor or acceptor were pre-incubated separately during 2 hours at 4°C with 0, 20, 50 and 100 µM of each compound, and then mixed as indicated above. Cell extracts were incubated with 1% DMSO alone for control.

Compound 14 is not a DNA intercalating agent (Ethidium bromide exclusion assay)

5µg of DNA (Calf-Thymus DNA, Sigma-Aldrich, St Louis, Mo, USA) and 1µg of Ethidium Bromide at 0.1mg/ml (EtBr, Sigma-Aldrich, St Louis, Mo, USA) were mixed together in milliQ water to obtain a volume of 50µl per well, in a black 96 well plate. The mixture was incubated for 15 min at room temperature (RT), protected from light, to allow the formation of the DNA/EtBr complex. Compound 14 and control compounds were diluted in milliQ water at different concentrations (25, 50, 100, 200µM) and added to the mixture to achieve the desired concentration (total volume = 100µl). After 1 hour incubation at RT in the dark, the DNA/EtBr complex was excited at 560nm and the fluorescence emission was recorded at 590 nm by using an OD reader (Fluostar Optima, Bmg Labtech, Ortenberg, Germany). Fluorescence values were normalized to wells containing only CT-DNA and EtBr. Doxorubicin (Teva classics, Pharmachemie b.v., Haarlem, Pays-Bas) was used as a positive control, while Docetaxel (Sanofi, Paris, France) and Gemzar (Lilly, Neuilly-sur-Seine, France) were employed as negative controls. Fluorescence of each compound alone was also tested at 200µM for the presence of background noise. Experiments were performed in triplicate.

After the promising results of compound 14 on the Hsp27/eIFE4 interaction, we performed a DNA binding assay to confirm that this compound was not a DNA intercalating agent potentially acting on DNA synthesis. We measured the fluorescence of DNA/Ethidium Bromide (BET) complex with and without our compound. We used Docetaxel and Gemzar as negative controls and Doxorubicin as a positive control, respectively. As represented in Supplementary Figure 1, we observed that phenazine compound 14 did not show any inhibition of the fluorescence of DNA/BET complex at all tested concentrations. In the contrary, Doxorubicin removed BET from DNA, thereby decreasing fluorescence. In parallel, the auto-fluorescence of the compound was tested at the highest dose, and no interference with the assay was detected. Our results allowed us to conclude that compound 14 was not a DNA intercalating agent.

Hsp27 mutant plasmids

Hsp27 deletion mutant plasmids (pcDNA4-His-Hsp27 WT, -Hsp27 N1, -Hsp27 N2, -Hsp27 C1, -Hsp27 C2) were a gift from Edward O'Brien (University of Ottawa Heart Institute, Canada) [27]. Hsp27 phosphorylation mutant plasmids (pENTR-Hsp27 WT, -Hsp27 3A, -Hsp27 3D) were donated by William Gerthoffer (University of Nevada School of Medicine, USA) [27]. LR gateway recombination reactions were performed between pENTR and pDEST26 (Invitrogen) destination plasmids, according to manufacturer's instructions (Invitrogen). All plasmids contained a histidine tag, merged to the proteins when produced, allowing the differentiation between endogenous and transfected proteins.

Chemical compounds

Phenazine derivatives were obtained from Michel Camplo and Olivier Siri (CINaM, Marseille, France) and resuspended in DMSO. Docetaxel was obtained from Sanofi-aventis (Paris, France) and stock solution of docetaxel was prepared with DMSO to the required concentrations before each experiment.

Treatment of cells with chemical compounds

PC-3 and PC ^{DR} cells were seeded into 10-cm dishes (1 250 000cells/well) or 12-well plates (50 000 to 100 000cells/well) according to the different experiments. One day after seeding, medium was changed to a new one containing DMSO (control) or compound 14 at different concentrations (25, 50, 100µM), according to the different experiments. Effects of the treatment were analyzed 48h later. The REG cells were transiently transfected with Hsp27 deletion mutant plasmids for 48h and treated after this with docetaxel (1nM) and the same protocol was used for transfection of Hsp27 phosphorylation mutant plasmids, treated with docetaxel (10 nM) in serum-free media (mimics androgen withdrawal *in vitro*).

Molecular modeling studies

Compound 14 was parameterized according to a consolidated procedure [1-4]. The unavailable 3D model structure of the full length wild-type (WT) Hsp27 was built and optimized by homology modeling/refining techniques [5]. The models of the three Hsp27 truncated

variants were obtained by deleting the relevant amino acid sequences, and optimizing the corresponding isoforms via energy relaxation/MD simulations in solution. The same protocol was employed to optimize the available 3D model of eIF4E (5BXV.pdb).

The docking modes of eIF4E onto WT Hsp27 and its three truncated isoforms were determined and optimized as described in our previous work [6, 7]. The relaxed Hsp27/eIF4E complexes were subjected to full-solvation molecular dynamics (MD) simulations, exploiting the last 100 ns of the MD data collection for the calculation of the binding free energy between the two proteins via the MM/PBSA (Molecular Mechanics/Poisson Boltzmann Surface Area) approach [8].

To study the mechanism of interference exerted on eIF4E/Hsp27 binding, compound 14 was docked into the eIF4E binding pocket, and the MM/PBSA scoring was again applied to estimate the affinity of 14 for eIF4E.

Finally, the ternary complex Hsp27/14/eIF4E was built by docking the MD Hsp27 model onto the last frame of the eIF4E/14 complex extracted from the corresponding equilibrated MD trajectory. The whole computational procedure was then applied again to study the binding energetics of the protein/drug/protein ensemble (see SI for full details). All simulations were carried out using the *Pmemd* modules of Amber 14 [9], running on a hybrid CPU/GPU calculation cluster.

Optimization of compound 14

The Amber ff14SB force field (Maier et al 2015) was used to parametrize all protein structures. The atomic partial charges for 14 were obtained using the RESP procedure (Bayly et al 1993), the electrostatic potentials being produced by single-point quantum mechanical calculations at the Hartree- Fock level with a 6-31G* basis set, using the Merz-Singh-Kollman van der Waals parameters (Besler et al 1990). Eventual missing force field parameters for the inhibitor molecule were generated using the Antechamber tool of Amber 14 (Case et al 2014) and the general AMBER force field (GAFF) (Wang et al 2010) for rational drug design.

Building and refinement of Hsp27/eIF4E complexes

The Hsp27/eIF4E protein-protein interface and the docking modes of eIF4E onto WT Hsp27 and its three truncated isoforms were determined using the HADDOCK server [10]. The resulting protein/protein docked conformations were clustered and visualized; then, in the absence of any relevant crystallographic information, the structure of each complex characterized by the lowest interaction energy in the prevailing cluster was selected for further modeling [7]. The selected Hsp27/eIF4E complexes were then solvated in a TIP3P [11] water box and, then, the required amount of Na^+ and Cl^- ions were added to neutralize the system and to mimic physiological salt conditions (150 mM), removing eventual overlapping water molecules. The solvated systems were subjected to a combination of steepest descent/conjugate gradient minimization of the potential energy, during which all bad contacts were relieved. The relaxed systems were then gradually heated to 300 K in three intervals by running constant volume-constant temperature (NVT) MD simulation, allowing a 0.5 ns interval per 100 K. Subsequently, 40 ns MD simulations under isobaric-isothermal (NPT) conditions were conducted to fully equilibrate each solvated compound. The *SHAKE* algorithm with a geometric tolerance of $5x10^{-4}$ Å was imposed on all covalent bonds involving hydrogen atoms. Temperature control was achieved using the Langevin temperature equilibration scheme and an integration time step of 2 fs. The particle mesh Ewald (PME) method [12] was used to treat the long-range electrostatics. At this point, these MD runs were followed by other 200 ns of NVT MD simulation.

eIF4E purification

eIF4E was cloned into pT7, the proteins were expressed in Bl21 STAR strain in LB in the presence of 100µg/mL ampicillin. Cultures were inoculated at 37 °C and grown to an OD_{600} of 0.9 at 37 °C, then induced with 0.5 mM Isopropyl-β-D-thio-galactoside (IPTG) and grown overnight at 24°C. Cells were harvested by centrifugation, cell pellets were resuspended in buffer (hepes 50mM [pH 7.6], 100 mM NaCl, 1mM EDTA, 1 mM dithiothreitol [DTT] and complete with protease Inhibitor Cocktail Tablets. Cells were lysed by a Sonicate and the lysate was cleared by centrifugation at 1h, 13000rpm. During spinning, equilibrate ~2ml of m^7GDP resin with the Elution wash buffer (Hepes 50mM (pH 7.5), KCl 2M, EDTA 1mM+DTT 1mM). The cleaved complexes were eluted with the same buffer. Concentrate to 1 ml and dialysis o/n into 50mM Phosphate pH 7.4, 50 mM NaCl, 1mM DTT. Then, add 1ml of 50 mM Phosphate buffer to dilute salt to 25mM and pass through ion exchange (MonoQ; salt gradient from 25 mM to 1 M NaCl). The gel-filtered protein complex was further purified by ion exchange on Resource Q (GE Healthcare) with a gradient from 150 mM to 1M NaCl.

ITC studies on the binding of eIF4E/14 complex

Isothermal titration calorimetry (ITC) experiments were performed with a MicroCal PEAQ-ITC calorimeter (Malvern, UK) at 25°C (cell volume = 280 µL). Thermodynamics of eIF4E/14 complex formation was investigated in HEPES buffered solutions at pH=7.

Specifically, a solution of eIF4E (25μM, sample cell) was titrated with 19 step-by-step injections of 2μL volume of 14 (250 μM, syringe). Solutions and buffer were degassed for 30 min at room temperature under stirring at 750 rpm prior to each experiment.

Molecular mechanics/poisson-boltzmann (MM/PBSA) theory

According to the MM/PBSA theory, the free energy of binding (ΔG_{bind}) between eIF4E and each Hsp27 variant can be calculated as the sum of different energetic contributions, corresponding to the average MD energies ($\Delta E_{MM} = \Delta E_{ele} + \Delta E_{vdW}$), the average solvation free energy ($\Delta G_{solv} = \Delta G_{solv,pol} + \Delta G_{solv,nonpol}$), and the entropic contribution ($-T\Delta S$). To further estimate the relative contribution of the residues involved in the protein/protein binding interface to the overall binding event, the enthalpic term of the ΔG_{bind} was deconvoluted into its main components for each individual residues ($\Delta H_{bind,res}$) of the proteins in the corresponding systems. This analysis was carried out using the MM/GBSA approach, [13, 14] and was based on the same snapshots considered for binding free energy calculation.

Protein-protein free energy of binding

To study the mechanism of interference exerted on eIF4E/Hsp27 binding, the optimized structure of compound 14 was initially docked into the retrieved eIF4E binding pocket by exploiting Autodock 4.3/Autodock Tools 1.4.6 (Morris et al 2009). The same cluster analysis, complex optimization, MM/PBSA and MM/GBSA scoring recipes described above for the eIF4E/Hsp27 ensemble was the applied to estimate the affinity of 14 for eIF4E.

Interference of compound 14 with the Hsp27/eIF4E complex formation

Finally, the ternary complex Hsp27/14/eIF4E was built by docking the MD Hsp27 model onto the last frame of the eIF4E/14 complex extracted from the corresponding equilibrated MD trajectory. The whole computational procedure was then applied again to study the binding energetics of the protein/drug/protein ensemble.

REFERENCES

1. Brambilla L, Genini D, Laurini E, Merulla J, Perez L, Fermeglia M, Carbone GM, Pricl S, Catapano CV. Hitting the right spot: mechanism of action of OPB-31121, a novel and potent inhibitor of the signal transducer and activator of transcription 3 (STAT3). Mol Oncol. 2015; 9: 1194-1206.
2. Pricl S, Cortelazzi B, Dal Col V, Marson D, Laurini E, Fermeglia M, Licitra L, Pilotti S, Bossi P, Perrone F. Smoothened (SMO) receptor mutations dictate resistance to vismodegib in basal cell carcinoma. Mol Oncol. 2015; 9: 389-397.
3. Gibbons DL, Pricl S, Posocco P, Laurini E, Fermeglia M, Sun H, Talpaz M, Donato N, Quintas-Cardama A. Molecular dynamics reveal BCR-ABL1 polymutants as a unique mechanism of resistance to PAN-BCR-ABL1 kinase inhibitor therapy. Proc Natl Acad Sci. 2014; 111: 3550-3555.
4. Laurini E, Posocco P, Fermeglia M, Gibbons DL, Quintas-Cardama A, Pricl S. Through the open door: preferential binding of dasatinib to the active form of BCR-ABLunveiled by in silico experiments. Mol Oncol. 2013; 7: 968-975.
5. Laurini E, Dal Col V, Mamolo MG, Zampieri D, Posocco P, Fermeglia M, Vio L, Pricl P. Homology model and docking-based virtual screening for ligands of the σ1 receptor. ACS Med Chem Lett. 2011; 2: 834-839.
6. Morgan A, Gandin I, Belcaro C, Palumbo P, Palumbo O, Biamino E, Dal Col V, Laurini E, Pricl S, Bosco P, Carella M, Ferrero GB, Romano C, et al. Target sequencing approach intended to discover new mutations in non-syndromic intellectual disability. Mutat Res. 2015; 781: 32-36.
7. Bozzi F, Conca E, Laurini E, Posocco P, Lo Sardo A, Jocolle G, Sanfilippo R, Gronchi A, Perrone F, Tamborini E, Pelosi G, Pierotti MA, Maestro R, et al. In vitro and in silico studies of MDM2/MDMX isoforms predict Nutlin-3A sensitivity in well/de-differentiated liposarcomas. Lab. Invest, 2013; 93: 1232-1240.
8. Massova I, Kollman PA. Combined molecular mechanical and continuum solvent approach (MM-PBSA/GBSA) to predict ligand binding. Perspect Drug Discov Des. 2013; 18: 113-135.
9. Case DA, Babin V, Berryman JT, Betz RM, Cai Q, Cerutti DS, Cheatham TE III, Darden TA, Duke RE, Gohlke H, Goetz AW, Gusarov S, Homeyer N, et al. AMBER 14. University of California, San Francisco. 2014.
10. Dominguez CR, Bonvin AM. HADDOCK: a protein-protein docking approach based on biochemical and/or biophysical information. J Am Chem Soc. 2003; 125: 1731-1737.
11. Jorgensen WL, Chandrasekhar J, Madura JD, Impey RW, Klein ML. Comparison of simple potential functions for simulating liquid water. J Chem Phys. 1983; 79: 926-935.
12. Toukmaji A, Sagui C, Board J, Darden T. Efficient particle-mesh Ewald based approach to fixed and induced dipolar interactions. J Chem Phys. 2000; 113: 10913-10927.
13. Onufriev A, Bashford D, Case DA. Modification of the generalized born model suitable for macromolecules. J Phys Chem B. 2000; 104: 3712-3720.
14. Tsui V, Case DA. Theory and applications of the generalized Born solvation model in macromolecular simulations. Biopolymers. 2000; 56: 275-291.

a

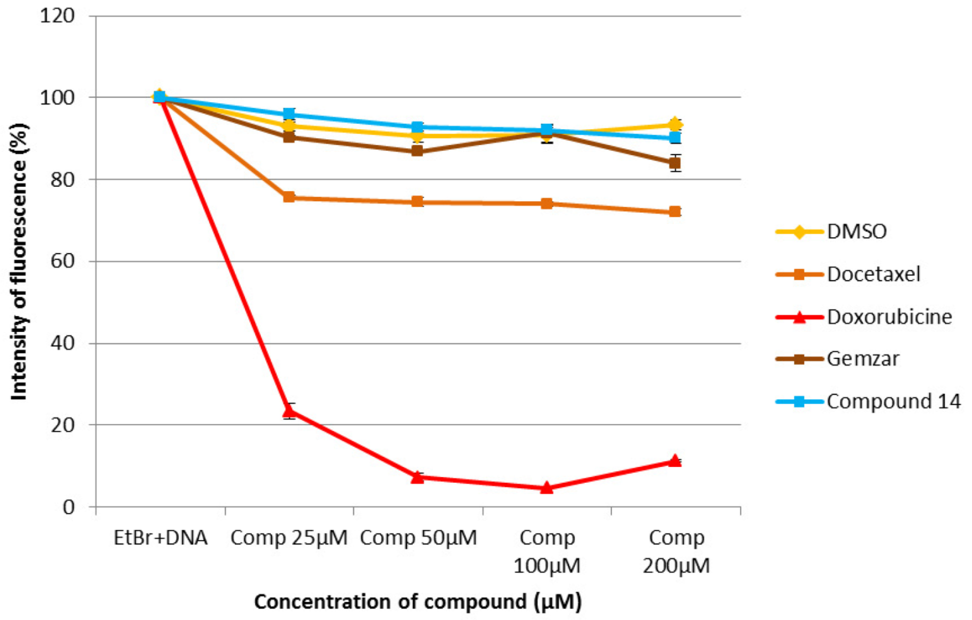

b

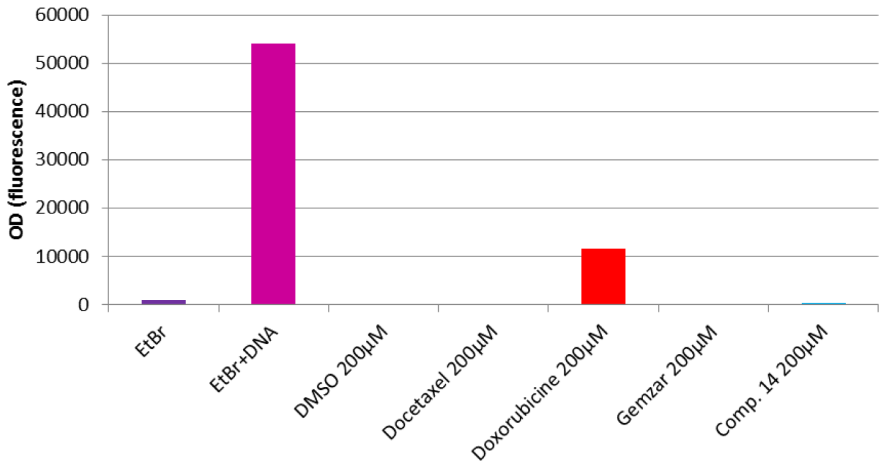

Supplementary Figure 1: Compound 14 is not a DNA intercalating agent. (a) 5µg of DNA and 1µg of Ethidium Bromide at 0.1mg/ml were mixed together in water and incubated for 15 min at RT to allow the formation of the DNA/EtBr complex. Compound 14 and control compounds were diluted in milliQ water at different concentrations (25, 50, 100, 200µM) and added to the mix to achieve the desired concentration and. After 1hour incubation at RT, the DNA/EtBr complex was excited at 560nm and the fluorescence emission was recorded at 590 nm by using an OD reader. The fluorescence values were normalized to wells containing only DNA and EtBr. **(b)** Fluorescence of each compound alone, measured by optic density (OD), has also been tested at 200µM to test the presence of a background noise that can disturb the analysis.
